# Exploring the effects of pandemics on transportation through correlations and deep learning techniques

**DOI:** 10.1007/s11042-023-15803-1

**Published:** 2023-06-07

**Authors:** Samah A. Gamel, Esraa Hassan, Nora El-Rashidy, Fatma M. Talaat

**Affiliations:** 1 Faculty of Engineering, Horus University, Damietta, Egypt; 2 Faculty of Artificial Intelligence, Kafrelsheikh University, Kafrelsheikh, Egypt

**Keywords:** Prediction, Deep Learning (DL), COVID-19, Convolutional Neural Network (CNN)

## Abstract

The COVID-19 pandemic has had a significant impact on human migration worldwide, affecting transportation patterns in cities. Many cities have issued "stay-at-home" orders during the outbreak, causing commuters to change their usual modes of transportation. For example, some transit/bus passengers have switched to driving or car-sharing. As a result, urban traffic congestion patterns have changed dramatically, and understanding these changes is crucial for effective emergency traffic management and control efforts. While previous studies have focused on natural disasters or major accidents, only a few have examined pandemic-related traffic congestion patterns. This paper uses correlations and machine learning techniques to analyze the relationship between COVID-19 and transportation. The authors simulated traffic models for five different networks and proposed a Traffic Prediction Technique (TPT), which includes an Impact Calculation Methodology that uses Pearson's Correlation Coefficient and Linear Regression, as well as a Traffic Prediction Module (TPM). The paper's main contribution is the introduction of the TPM, which uses Convolutional Neural Network to predict the impact of COVID-19 on transportation. The results indicate a strong correlation between the spread of COVID-19 and transportation patterns, and the CNN has a high accuracy rate in predicting these impacts.

## Introduction

The global effect of the new coronavirus 2019 (COVID-19) pandemic has been significant. According to the most recent data from the World Health Organization (WHO), as of 19 May 2021, more than 146,4 million confirmed cases of COVID-19, including 3,4 million fatalities, have been reported globally. During the COVID-19 pandemic, transportation networks are critical for curbing the virus's spread and facilitating the restart of productivity. Many governments and localities issued "stay-at-home" orders early in the pandemic to limit and stop the virus's spread. For Example, the authorities of Wuhan enacted outward travel limits at an early stage, to prevent the virus from spreading. According to studies, the travel restriction in Wuhan and the national emergency response slowed the spread of the pandemic and eventually limited the impact of COVID-19 in China. The shutdown of Wuhan is estimated to have slowed the spread of the pandemic to adjacent cities by 2.91 days [35]. In the post COVID-19 period, travel and traffic practices have changed.

At the beginning of 2020, the COVID-19 pandemic turned such a terrible infection that the global began to close. Meanwhile, there are a considerable number of personal vehicles in the present transport system. For example, in the United States, there were 286.9 million registered vehicles in 2020. In front of these data, traffic congestion, fuel consumption and emissions from greenhouse gas have become a critical problem. According to data from the American Transportation Institute, traffic congestion cost US more than $74.1 billion annually [8]. In 2018, Energy Information Administration in United States reveals data that 28.2% of the total U.S. energy uses were in the transportation sector and 28% of fuel emissions.

Furthermore, Transport networks also help in economic reopening. The Traffic Transportation System serves as a support to restart work and manufacturing when the pandemic had been contained. Since May 2020, the governments of the United States and several European nations have reduced their stay-at-home orders and progressively eased some travel restrictions. Understanding empirical traffic congestion patterns is essential for improving the management and control of non-recurring traffic conditions during and after the COVID-19 pandemic [26].

Previous research has focused on the patterns of evacuation congestion during natural disasters or severe weather, such as hurricanes and tornadoes [17]. However, due to the rarity of pandemics, there is limited empirical research on traffic congestion patterns during and after a pandemic. There are significant distinctions between natural disasters and pandemics [19]. Natural catastrophes have a relatively limited impact, usually a few weeks or months, whereas pandemics have an impact that can last for months or even years. Furthermore, traffic needs or demands during natural catastrophes differ significantly from those during pandemics. When it comes to natural disasters, the most pressing worry is evacuating people from susceptible places in a timely manner. On the other hand, during and after a pandemic, demands for traffic are more complex and change over time. It is critical to stop the virus from spreading by limits the transportation system. As well, following the onset of a pandemic, inhabitants' travel habits will vary due to a variety of circumstances [3, 6]. With the unusual conditions of a pandemic and the concurrent impact of various factors, the patterns of traffic situations variation are complicated and difficult to predict [10, 16].

**Fig. 1:**
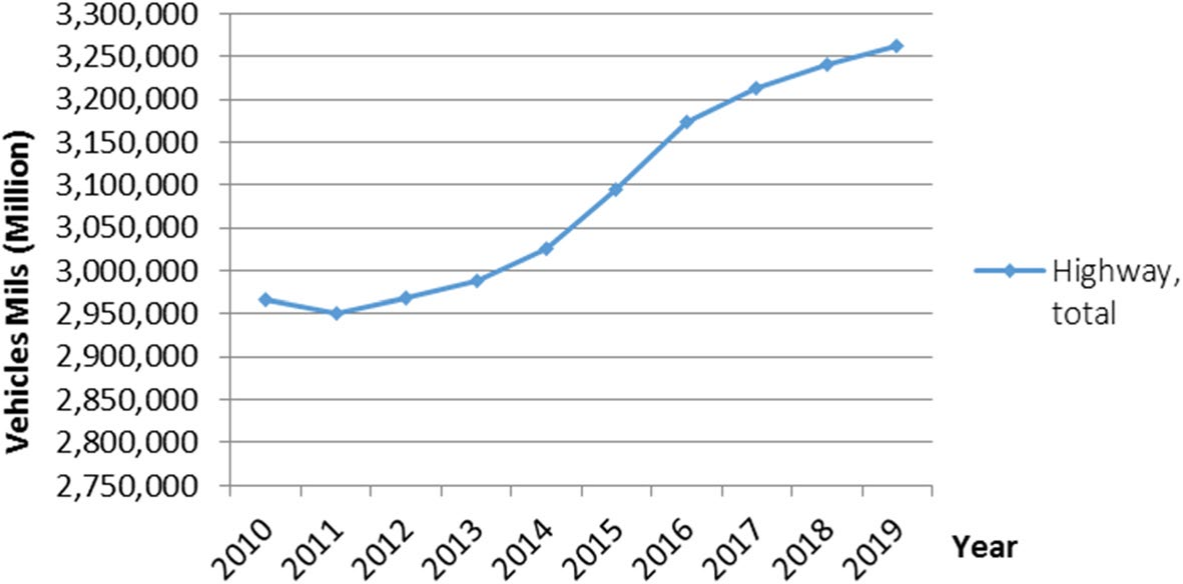
Total number of vehicles travelling on highways

Figure 1 illustrates how the total number of cars used on highways increased on average by 1% year from 2000 to 2018, rising to more than 3.26 (millions), up from 2.96 (millions), according to statistics from the Bureau of Transportation Statistics (BTS) [1].

**Fig. 2:**
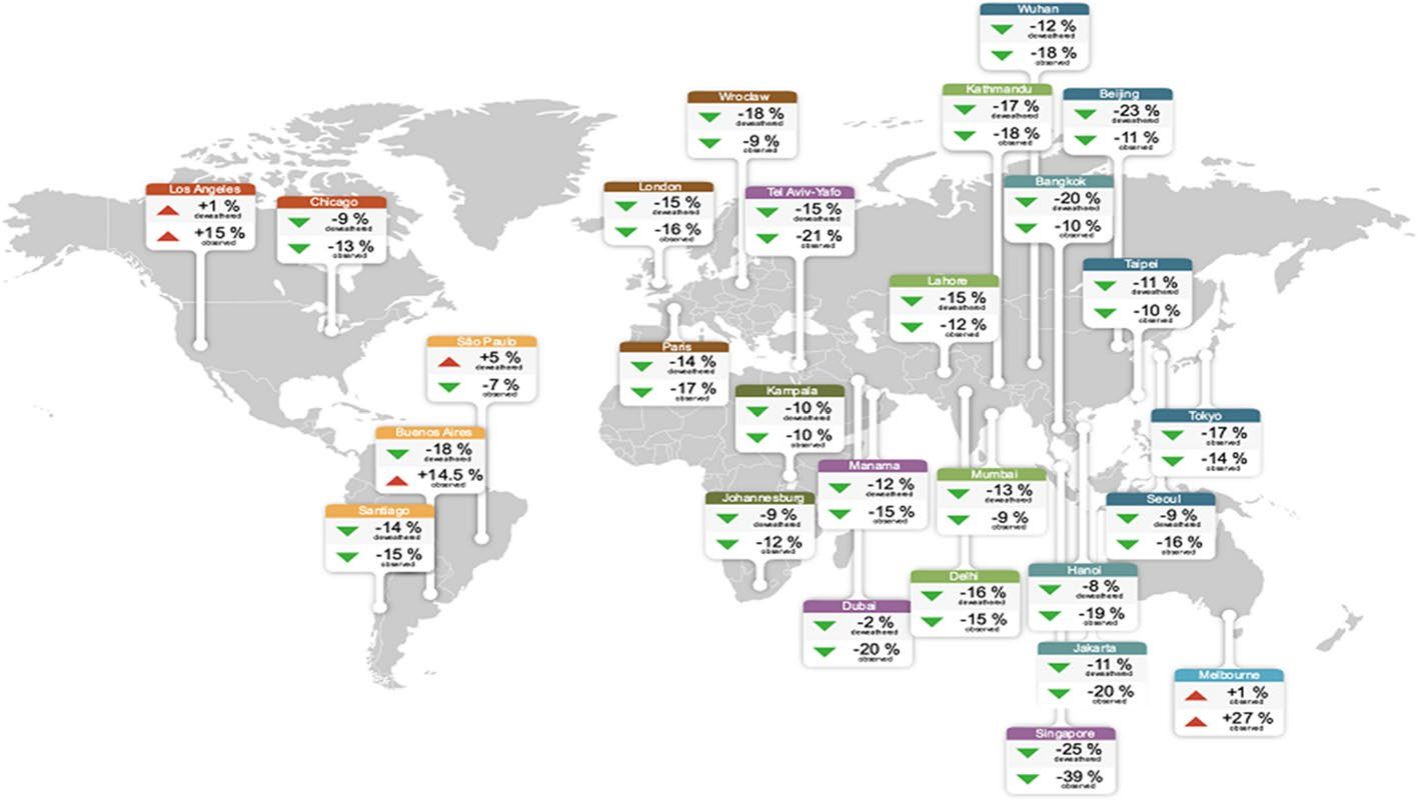
Analyze air quality and map for major cities (https://www.iqair.com/usa)

A Traffic Dashboard was launched by A Michigan-based Transportation Company for data management, MS2, to present up-to-date information for observing the impacts. The daily traffic volume trends (DTVT) is a metric about the everyday traffic volume variation compared to the same day of the week in the same month for the most recent years, it was developed and published to exhibit the traffic volume variations across the United States [22]. Based on their data, overall national traffic has been reduced by near to 65%. Google offered similar statistics [11]. Consequently, the decrease in traffic volumes by the State was between 40 and 65%. As the volume of traffic was dramatically changing, the air quality in the Los Angeles region for example was significantly improved (https://www.iqair.com/usa). Also, the countries with historically level of pollution with higher than PM2.5 have seen decline reduced when locking has taken place as shown in Fig. 2.

Crowd computing can simplify city traffic evaluation, which is expensive to audit and diagnose structurally. Results appear satisfactory and trustworthy, able to alter for unique needs or improvement. Mobile sensors help reduce external factors such as involuntary user movement, measurement, and coverage mistakes. However, the intangible metrics and structures used in indicator analysis can be used to build sustainability assessing indicators that can be used in general-purpose sustainability assessment frameworks [28].

The aim of this paper is to analyze the relation between COVID-19’s and the transportation sector using correlations and using machine learning techniques. The traffic model for five different networks was simulated. This paper introduces a proposed Traffic Prediction Technique (TPT). The TPT is composed of two main methodologies which are: (i) Methodology of impact calculation which uses Pearson’s Correlation Coefficient (PCC), and Linear Regression (LR), (ii) Traffic Prediction Module (TPM).

The rest of this paper is arranged in the following manner: Section 2 introduces some concepts in: (i) Spreading of Virus, (ii) Economic Implications, (iii) Travel Demand and Related Impacts, and (iv) Convolutional Neural Networks (CNNs). A brief summary of the research that is relevant to this topic is provided in Section 3. The study area, dataset, and research technique are detailed in Section 4. The evaluation results and discussion are introduced in Section 5. The final section, Section 6, presents the conclusion.

### Research gap

Earlier studies on this topic focused on natural disasters or major accidents, but only a few studies looked into the empirical patterns of pandemic traffic congestion. There is a lack of research on using machine learning techniques to analyze the impact of COVID-19 on the transportation sector.

The main contributions of this paper are:

- Analyzing the relation between COVID-19 and the transportation sector using correlations and machine learning techniques.
- Introducing a proposed Traffic Prediction Technique (TPT) that uses both Pearson’s Correlation Coefficient (PCC) and Linear Regression (LR) in the Methodology of impact calculation, and a Traffic Prediction Module (TPM) that utilizes Convolutional Neural Network (CNN) to predict the impact of COVID-19 on transportation.
- Simulating the traffic model for five different networks and showing that there is a strong correlation between the spread of COVID-19 virus and transportation.
- Demonstrating that CNN has a high accuracy rate in predicting the impact of the spread of COVID-19 virus on transportation.

## Background

This section introduces some concepts which are: (i) Spreading of Virus, (ii) Economic Implications, (iii) Travel Demand and Related Impacts, and (iv) Convolutional Neural Networks (CNNs).

### Spreading of virus

The spread of a pandemic such as COVID-19 is aided by the connectivity of our global transportation networks. Due to the global nature of transportation, the virus rapidly spread from a single neighborhood to other parts of the world, wreaking havoc on local communities. Thus, it continued for a very long time. In the context of disease transmission, transportation can be considered a "disease vector" because it can disseminate diseases via at least three mechanisms: (i) Infected individuals and objects travel to other regions, where they can transmit the disease. People using public transportation tend to congregate in groups and at higher densities, increasing the likelihood that infected passengers will infect other passengers.

In this field, there are a number of research questions to consider, including: (i) what role does transportation play in the propagation of pandemics like COVID-19? (ii) How can we simulate the spread of a pandemic and assess the efficacy of various mitigation strategies and policies? (iii) How successful are cleaning chemicals and different surfaces at reducing pathogens? (iv) To prevent the spread of disease, what behavioral and structural adjustments may be made to passenger interactions while in transit?

### Economic implications

The globe has taken significant measures to stop COVID-19 from spreading. However, these behaviors have far-reaching ramifications, including direct economic effects. Apart from the fact that a huge segment of the population will be unable to work, the complete implosion of travel during the epidemic, as well as possible decreases afterward, has numerous long-term economic ramifications. Large losses in revenue earned by fuel tax and sales tax are one example. This compounds the problems that transportation agencies are already dealing with as a result of the transition to more fuel-efficient vehicles, such as hybridization and electrification.

Reduced travel will also lead to a decrease in fees for parking, tolls, penalties, and other sources of income. Significant drops in state highway and transportation earnings have already been recorded by some state transportation agencies. As a result, financing for new transportation projects, transportation system maintenance, and public transportation services will be significantly reduced. The drop in oil prices has a number of economic consequences, including employment losses, and adoption of more fuel-efficient vehicles, such as hybrids and fully electric vehicles, may be hindered.

Airlines, airports, producers of planes, dealers in cars, cruise lines, public transit, and privately owned cars for rent are among the transportation-related companies that are struggling during COVID-19 (taxis, Uber, Lyft, etc.). During these eras, the seaport and trucking industries appear to be thriving. There are several research questions to examine in this topic, including: (i) what are the potential ramifications for transportation income, and what alternative revenue-generating possibilities are available? (ii) is it possible to charge and pay for transportation infrastructure and services in a more efficient way? (iii) How can we account for the true cost of traveling and transporting goods, as well as the impact of negative externalities? (iv) What influence does the lowering oil price have on transportation in general, and what are the potential negative consequences for the adoption of more fuel-efficient vehicles like hybrids and completely electric vehicles? (v) What sorts of transportation industries are resilient in the face of a pandemic, and which do not? (vi) How can the transportation sectors, which are particularly vulnerable, be made more viable in these circumstances?

### Travel demand and related impacts

The workplace culture will almost undoubtedly undergo a long-term shift. People whose jobs require them to predominantly use a telephone, computer, and internet connection are not required to report to the office every day. To save money on real estate, some businesses may opt to have their employees work permanently from home. Consequently, there will likely be a substantial shift toward enabling more employees to work remotely. This will free up a substantial amount of commute time (car miles traveled), thereby reducing traffic congestion, traffic accidents, vehicle emissions, pollution, and a variety of other negative externalities associated with transportation [9].

The huge improvement in air quality due to the significant reduction in travel during the COVID-19 outbreak is one evident example. We will see major health benefits if these lower levels persist. Preexisting respiratory diseases are more common in places with high PM 2.5 levels, resulting in greater fatality rates. This will change the way we think about air quality during and after a pandemic. The significant decline in traffic accidents and fatalities is another trend of note. However, fatalities still occur due to increased racing during low-traffic conditions, and in a few states, traffic-related fatalities have increased. In this field, there are numerous research questions to consider, including: (i) If telecommuting becomes the norm, what effect will it have on travel? (ii) How will short-, medium-, and long-term travel demand vary by mode? What impact would reduce travel have on issues such as traffic safety, vehicle emissions, congestion, road maintenance, pollution, and public health? Will society adopt a new approach to issues such as air quality, automobile accidents, and noise?

### Convolutional Neural Networks (CNNs)

A Convolutional Neural Network (ConvNet/CNN) [2] is a Deep Learning (DL) technique that can take an input features, assign value to various aspects/objects in the input data, and distinguish between them. The amount of pre-processing required by a ConvNet is substantially less than that required by other classification techniques. ConvNets have the capacity to learn these filters/characteristics, despite the fact that they are hand-engineered in elementary systems. CNN is a type of feed-forward Neural Network containing convolution layers and pooling processes [21].

## Related work

Prior research on non-recurring traffic patterns concentrated primarily on natural disasters and severe weather, including empirical examinations of traffic flow and travel behavior, as well as evacuation modeling and simulation. The authors of [5] evaluated the impacts of cumulative unit rainfall and cumulative unit snowfall on non-recurring traffic congestion per unit distance. Based on traffic data collected in Cape May, New Jersey, during Hurricane Irene, they examined an empirical evacuation reaction curve in [18]. Previous studies have concentrated on evacuation modeling and simulation, including both static and dynamic models, as well as actual travel behavior and traffic patterns. The authors of [23] provide an exhaustive evaluation (2013) of evacuation modeling and simulation over the past decade. Following the East Japan earthquake, the authors of [12] used GPS data from exploration vehicles and smart phones to analyze people's behavior and traffic congestion patterns. The findings were essential for appropriate evacuation planning. The authors in [14] studied the contributing variables to travelers' behavioral responses to pre-planned events using vehicle trajectory data.

During the COVID-19 pandemic, only a few studies examined the relationship between human movement and the dissemination of the virus. [4] To predict the impact of travel restrictions on viral transmission, a global model of population disease transmission was utilized. The data revealed that the Wuhan travel quarantine effectively slowed the overall trend of viral spread in mainland China by three to five days, and that case imports were reduced by more than 80 percent until mid-February. The authors of [10] established a system to capture variations in the spatiotemporal distribution of exposure and COVID-19 transmission risk based on human movement in Shanghai. [15] Performed a data-driven investigation of travel behaviors during the pandemic from the following perspectives: (1) check-in time at venues, (2) method of origin destination, (3) distance of origin, (4) mode of transportation, and, (5) categories of venues to be visited; this study provided decision-making support for a better understanding of COVID-19's effect on traffic activities, as well as more targeted epidemic preventive strategies.

While in [24] authors investigated the link between COVID-19 perception and travel risk perception and behavior among DACH travelers (Germany, Austria, and Switzerland). Over a short period of time, the results demonstrated a considerable rise in COVID-19 risk perception as well as travel risk perception. The socially optimal quarantine and travel (social activity) restriction policies for infectious viruses, such as COVID-19, were analyzed in [25]. Results indicate that government intervention is necessary to induce individual travelers to internalize the external costs of infection risks imposed on others and the health-care system. The authors of [23] analyzed the fundamental structure and application of numerous epidemic spread models and argued that more in-depth research is required to investigate the mutual feedback mechanism of epidemics and individual behavior. While the authors of [7] used GPS data from smart phones in the United States to illustrate how stay-at-home directives affect human mobility. At last authors in [20] look at how traffic congestion changed in Shanghai during and after the COVID-19 outbreak in terms of space and time. Based on Traffic Analysis Zones' average speed data (TAZs). As a result, there are enough research to prove that people' travel habits have changed as a result of the epidemic.

As stated previously, previous research has frequently centered on practical travel behavior and traffic flow analyses in the context of natural disasters or extreme weather. Due to the rarity of this occurrence, few studies have examined the actual characteristics of urban traffic flow during a pandemic. Although some recent research examined the relationship between human mobility and viral spread during the COVID-19 pandemic, the majority of studies focused on the risk of viral spread and the consequences of travel restrictions. The prior research on traffic flow is summarized in Table 1.

**Table 1:** Recent Studies on traffic behavior based on different factors

Authors	Year of publish	Methodology
Chung, Y. [5]	2012	Effects on non-recurrent traffic congestion per unit distance of cumulative unit rainfall and cumulative unit snowfall
Li, J. et al. [18]	2014	Empirical reaction curve for evacuation based on traffic data obtained in Cape May, New Jersey during Hurricane Irene
Murray-Tuite, P. et al. [23]	2013	Comprehensive assessment of evacuation modeling and simulation during the last decade
Hara, Y. et al. [12]	2015	Using GPS data from exploration vehicles and smartphones, a study of post-earthquake human behavior and traffic congestion patterns was conducted
Hu, X. et al. [14]	2019	Study of contributing variables to travelers' behavioral responses to pre-planned events using vehicle trajectory data
Chinazzi, M. et al. [4]	2020	Model of global population disease transmission to predict the impact of travel restrictions on viral transmission; specifically, Wuhan travel quarantine effectively slowed the overall trend of viral propagation in mainland China by three to five days
Gan, T. et al. [10]	2020	System to detect variations in the spatial and temporal distribution of exposure and COVID-19 transmission risk in Shanghai based on human movement
Huang, J. et al. [15]	2020	Data-driven investigation of travel behaviors during the COVID-19 pandemic, including check-in time at venues, manner of origin destination, distance between origin and destination, mode of transportation, and venue categories to be visited
Neuburger, L. et al. [24]	2020	Examining the relationship between COVID-19 perception, travel risk perception, and travel behavior among DACH (Germany, Austria, and Switzerland) travelers
Oum, T. H. et al. [25]	2020	Examining the socially optimal quarantine and travel (social activity) restriction policies for infectious viruses, such as COVID-19
Murray-Tuite, P. et al. [23]	2013	Analysis of fundamental structure and application of numerous epidemic spread models
Engle, S. et al. [7]	2020	Use of GPS data from smartphones in the United States to illustrate how stay-at-home directives affect human mobility
Jian Li et al. [20]	2021	Based on Traffic Analysis Zones' (TAZ) average speed data, an examination of how traffic congestion in Shanghai changed during and after the COVID-19 outbreak

We can summarize the major findings of prior studies as follows: i) Modelling and simulation of evacuation, including static and dynamic models ii) Following natural catastrophes, GPS data from exploration vehicles and smartphones have been analyzed to determine patterns of human behavior and traffic congestion; iii) During the COVID-19 pandemic, researchers examined the connection between human movement and the transmission of the virus. iv) Studies have analyzed travel behaviors during the pandemic, including check-in time at venues, mode of transportation, and categories of venues visited, v) Research has investigated the relationship between COVID-19 perception and travel risk perception and behavior among travelers, and vi) Some studies have examined how stay-at-home directives impact human mobility during the pandemic.

The research gap in the previous algorithms and strategies can be summarized as follows:

1. Lack of focus on pandemic-related traffic congestion: Previous studies on traffic prediction have primarily focused on natural disasters or major accidents, but few have looked at the impact of pandemics on transportation. This research gap limits the ability of traffic management systems to effectively respond to the unique challenges posed by pandemics.
2. Limited use of machine learning techniques: While some previous studies have used machine learning techniques for traffic prediction, the majority have relied on traditional statistical methods. This research gap limits the potential accuracy and effectiveness of traffic prediction models, especially in the context of complex and dynamic traffic patterns.
3. Incomplete data preparation and cleaning: Many previous studies have not adequately addressed the challenges of data preparation and cleaning, which can lead to inaccurate or biased predictions. This research gap highlights the need for more rigorous data processing methods to ensure the quality and reliability of traffic data.
4. Lack of integration with other traffic management systems: Previous algorithms and strategies have not fully exploited the potential of integrating traffic prediction models with other traffic management systems, such as intelligent transportation systems (ITS) and traffic signal control systems. This research gap limits the ability of traffic management systems to respond to real-time traffic conditions and optimize traffic flow in a coordinated manner.

This research aims to address the research gap by proposing a novel Traffic Prediction Technique (TPT) that incorporates machine learning techniques, particularly the use of Convolutional Neural Network (CNN), to predict the impact of the COVID-19 pandemic on the transportation sector. The proposed TPT is also composed of a methodology of impact calculation that utilizes Pearson’s Correlation Coefficient (PCC) and Linear Regression (LR). The integration of these techniques is expected to provide a more accurate and reliable prediction of traffic congestion during pandemic situations, which can aid in the development of effective traffic management and control strategies. Additionally, this research analyzes the empirical patterns of pandemic traffic congestion in five different networks, providing valuable insights into the relation between COVID-19 and transportation sector.

## The proposed Traffic Prediction Technique (TPT)

The proposed Traffic Prediction Technique (TPT) is a novel approach that uses a combination of correlation analysis, linear regression, and machine learning to predict the impact of the spread of COVID-19 on transportation networks. The TPT consists of two main methodologies:

(i). Methodology of impact calculation: This methodology is used to calculate the impact of COVID-19 on traffic congestion. It uses Pearson’s Correlation Coefficient (PCC) to measure the correlation between the number of COVID-19 cases and the level of traffic congestion. Linear regression is then used to model the relationship between the two variables and estimate the impact of COVID-19 on traffic congestion. This methodology is designed to identify the statistical significance of the relationship between COVID-19 cases and traffic congestion and provide a quantitative estimate of the impact.
(ii). Traffic Prediction Module (TPM): This module uses Convolutional Neural Network (CNN) to predict the impact of COVID-19 on traffic congestion in the future. The TPM takes as input a set of historical traffic data and COVID-19 case data and trains a CNN model to learn the complex relationships between the two variables. The model is then used to predict the impact of future COVID-19 cases on traffic congestion. The CNN model is chosen because it is particularly well-suited for analyzing sequential data such as traffic flow, and has been shown to outperform other machine learning algorithms in traffic prediction tasks.

The TPT is an original approach that combines traditional statistical methods with state-of-the-art machine learning techniques to predict the impact of COVID-19 on traffic congestion. The use of PCC and linear regression provides a rigorous statistical foundation for the analysis, while the CNN model provides a powerful tool for predicting future traffic patterns. Overall, the TPT has the potential to provide valuable insights into how the spread of COVID-19 is impacting transportation networks, and to aid in the development of effective traffic management strategies during the pandemic and beyond.

The proposed TPT combines five main phases: (i) Phase 1: Data Preparation and Cleaning; (a) Collect and preprocess data from various sources such as traffic cameras, weather stations, construction records, and special event schedules. (b) Identify and remove any missing, inconsistent, or erroneous data points. (c) Normalize the data to ensure that all features are on the same scale. (ii) Phase 2: Correlation and Regression Analysis; (a) Use Pearson’s Correlation Coefficient (PCC) to identify the correlation between COVID-19 cases and traffic congestion. (b) Perform linear regression analysis to determine the impact of COVID-19 cases on traffic congestion. (c) Evaluate the statistical significance of the results and adjust for any confounding factors.

(iii). Phase 3: Machine Learning Modeling; (a) Train and test multiple machine learning models, including CNN, RNN, and LSTM, to predict traffic congestion based on COVID-19 case data and other relevant factors. (b) Use feature selection techniques to identify the most important predictors of traffic congestion. (c) Optimize model hyperparameters to improve accuracy and generalization performance. (iv) Phase 4: Model Evaluation and Comparison; (a) Evaluate the performance of the CTIP algorithm using various metrics, such as mean absolute error (MAE), mean squared error (MSE), and coefficient of determination (R-squared). (b) Compare the performance of TPT to other approaches, such as traditional statistical models or other machine learning algorithms. (c) Provide a detailed analysis of the strengths and weaknesses of TPT and identify areas for future improvement. (v) Phase 5: Deployment and Integration; (a) Deploy TPT in a production environment, such as a traffic management center, to provide real-time traffic impact predictions. (b) Integrate TPT with other traffic management systems, such as intelligent transportation systems (ITS) and traffic signal control systems, to improve overall traffic management efficiency and effectiveness.

### Phase 1: Data preparation and cleaning

Data Preparation and Cleaning involves three main steps as depicted in Algorithm 1; (i) Step 1: Collect data from various sources; (a) Gather traffic data from cameras or other sensors. (b) Obtain weather data from weather stations. (c) Collect construction records and schedules of special events. (ii) Step 2: Preprocess the data; (a) Remove duplicates and irrelevant data. (b) Convert data to a common format (e.g., CSV, JSON). (c) Check data for consistency and accuracy. (d) Remove outliers and missing data. (iii) Step 3: Normalize the data; (a) Normalize the data to ensure that all features are on the same scale. (b) Use Min–Max normalization or Z-score normalization.

**Algorithm 1:**
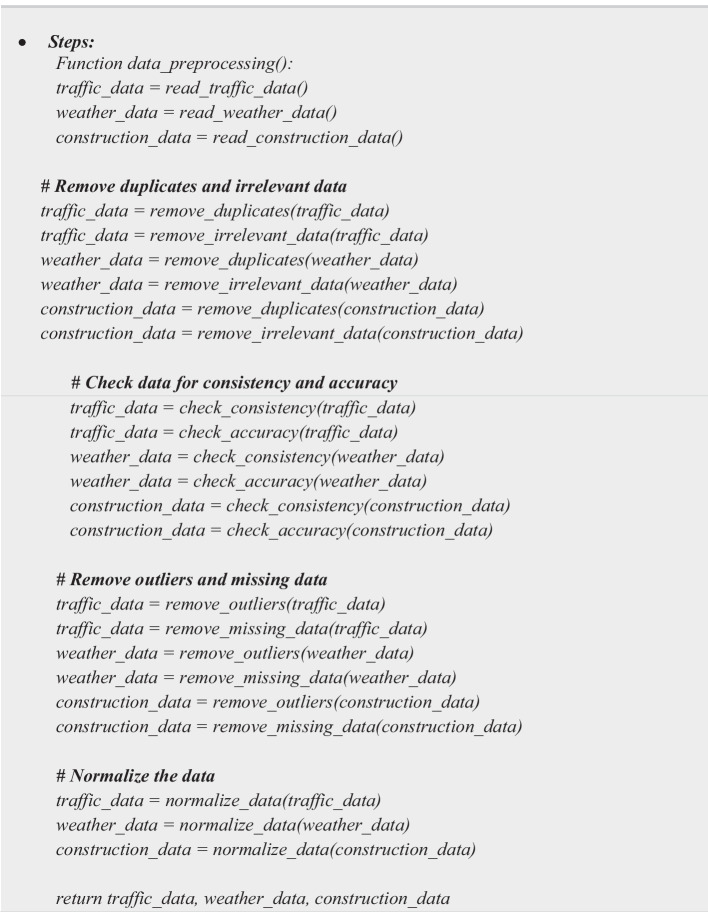
Data Preparation and Cleaning Algorithm

### Phase 2: Correlation and regression analysis

Correlation and Regression Analysis involves three main steps as depicted in Algorithm 2. (i) Step 1: Calculate the Pearson's Correlation Coefficient (PCC); (a) Calculate the PCC between COVID-19 cases and traffic congestion data. (b) Determine the strength and direction of the correlation. (ii) Step 2: Perform Linear Regression Analysis; (a) Use the traffic and COVID-19 data to perform linear regression analysis. (b) Determine the regression equation that best fits the data. (c) Calculate the regression coefficients and their significance. (d) Evaluate the goodness of fit of the regression model. (iii) Step 3: Evaluate the Statistical Significance; (a) Determine the statistical significance of the results using hypothesis testing. (b) Adjust for any confounding factors that may affect the results.

**Algorithm 2:**
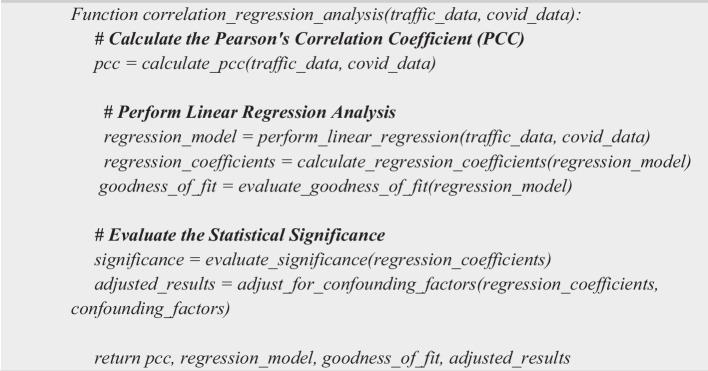
Correlation and Regression Analysis Algorithm

Correlation and Regression Analysis involves two methods: (i) Pearson’s Correlation Coefficient (PCC), and (ii) Linear Regression (LR).

#### Pearson’s Correlation Coefficient (PCC)

The correlation coefficient quantifies the degree of association between two variables. There are numerous types of correlation coefficients, with Pearson's being the most prevalent. In linear regression, Pearson's correlation (also known as Pearson's r) is a commonly employed correlation coefficient [33]. A correlation coefficient of 1 indicates that for each positive increase in one variable, the other variable also increases proportionally. For instance, shoe sizes increase nearly proportionally to foot length. A correlation coefficient of -1 indicates that for every increase in one variable, the other variable decreases proportionally. For example, the amount of petroleum in a vehicle's tank decreases in (near) perfect proportion to the vehicle's speed. Zero indicates that there is no increase for each increment, either positive or negative. The two have no relationship whatsoever. Pearson's formula for the correlation coefficient is one of the most utilized. To examine the impact of the spread of COVID-19 on traffic, we compute the Pearson's correlation coefficient (rt) between the Number of COVID-19 Cases (NC) and traffic (T), as depicted in Eq. (1).

1 $$\mathrm{rt}=\frac{n\left(\sum NCT\right)-(\sum NC)(\sum T)}{\sqrt{\left[n\sum{(NC)}^2-\left(\sum NC\right)^2\right]\left[n\sum{(T)}^2-\left(\sum T\right)^2\right]}}$$

#### Linear Regression (LR)

In the second stage, we used linear regression analysis to determine the extent to which the number of COVID-19 cases influences traffic. In statistical modeling, regression analysis is used to assess the relationships between two or more variables. Multiple R quantifies the strength of a linear relationship between two variables. R's absolute value indicates the intensity of the relationship. The relationship is more robust the larger the absolute value.

### Phase 3: Machine learning modeling

**Fig. 3:**
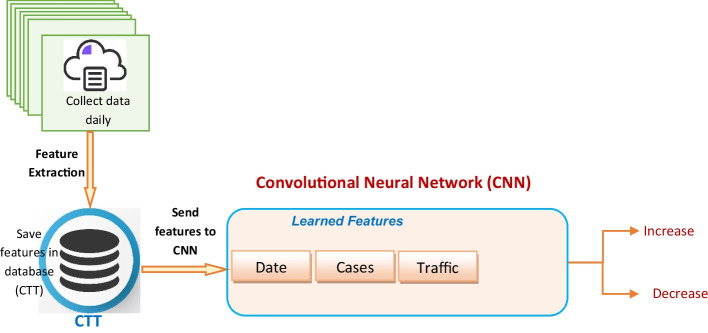
The EPM Steps


The Traffic Prediction Module (TPM) uses Convolutional Neural Network (CNN) to predict the **impact of the spread of COVID-19 virus on the transportation. The prediction process is done using** CNN as shown in Fig. 3 and in Algorithm 3.

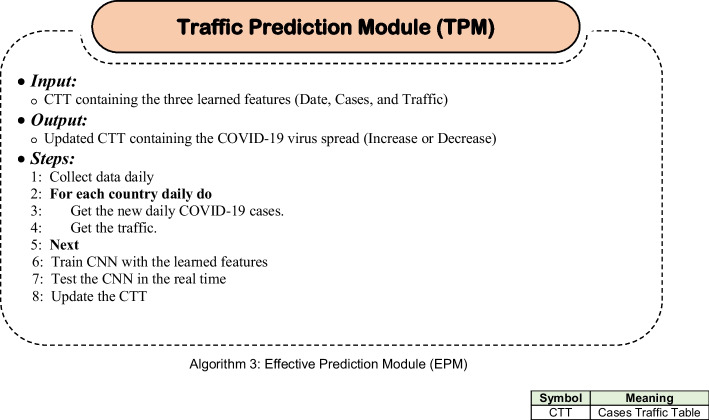


### Phase 4: Model evaluation and comparison

Model Evaluation and Comparison involves three main steps as depicted in Algorithm 4. (i) SStep 1: Evaluate the performance of the CTIP algorithm; (a) Use various metrics, such as mean absolute error (MAE), mean squared error (MSE), and coefficient of determination (R-squared) to evaluate the performance of the TPT algorithm. (b) Calculate the metrics using the predicted values and the actual values. (ii) Step 2: Compare the performance of TPT to other approaches; (a) Compare the performance of TPT to traditional statistical models or other machine learning algorithms. (b) Calculate the same metrics used in step 1 for the other approaches. (iii) Step 3: Analyze the strengths and weaknesses of TPT; (a) Provide a detailed analysis of the strengths and weaknesses of TPT based on the performance metrics and other factors, such as interpretability, scalability, and computational complexity. (b) Identify areas for future improvement and potential research directions.

**Algorithm 4:**
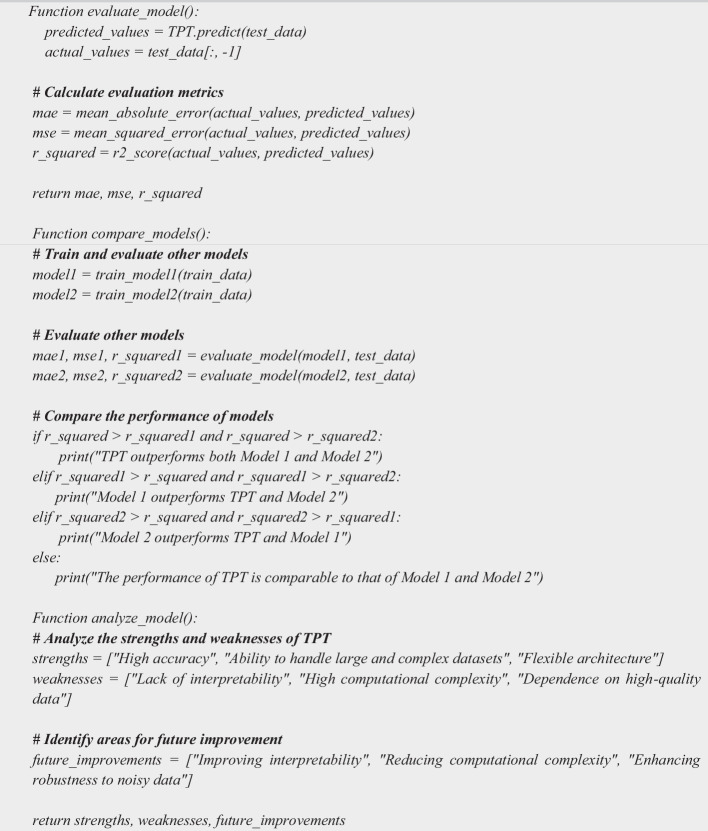
Model Evaluation and Comparison Algorithm

### Phase 5: Deployment and integration

Deployment and Integration involves two main steps as depicted in Algorithm 5. (i) Step 1: Deploy TPT in a production environment; (a) Install TPT in a traffic management center or other production environment. (b) Configure TPT to receive real-time traffic data from traffic cameras, sensors, and other sources. (c) Set up TPT to provide real-time traffic impact predictions. (ii) Step 2: Integrate TPT with other traffic management systems; (a) Identify other traffic management systems that could benefit from TPT's predictions. (b) Develop an integration plan for TPT and the other systems. (c) Configure TPT to work with the other systems. (d) Test the integration to ensure that TPT is providing accurate and useful predictions.

**Algorithm 5:**
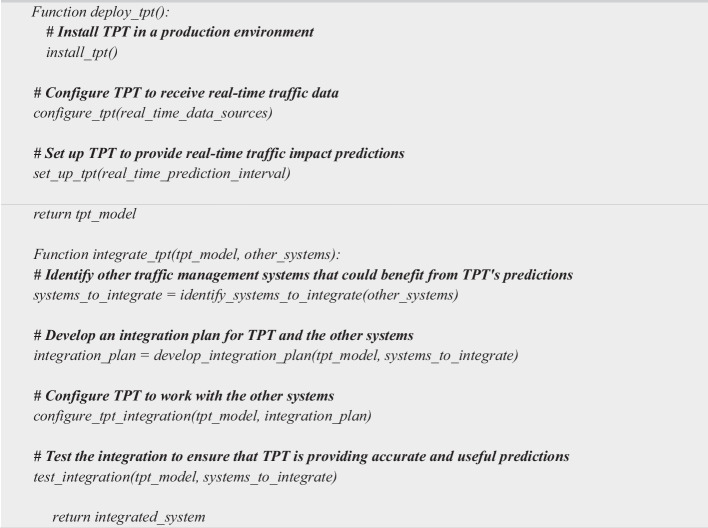
Deployment and Integration Algorithm

## Implementation and evaluation

This section presents the used dataset and the results of the methodology presented in Section 4, which was carried out to investigate how the COVID-19 impact on the traffic regulation system.

### Used dataset

In this paper, we use Dataset to illustrate the traffic in six different countries during the period from 2020–03-19 to 2021–09-25 and also illustrate the spread of COVID-19 virus (by analyzing number of cases each day) parallel with traffic on that day. The dataset for COVID-19 is from WHO. While, the dataset for traffic in each country is from Google mobility maps and Apple maps.

In this paper, the experimentation was carried out using Python programming language, and the libraries used include NumPy, Pandas, Scikit-learn, and TensorFlow.

Firstly, the data for traffic and COVID-19 cases were collected from the respective sources and preprocessed to remove any missing values or outliers. Then, Pearson correlation coefficient was used to analyze the relationship between traffic and COVID-19 cases.

Next, linear regression was used to model the relationship between traffic and COVID-19 cases. The data was split into a training dataset and testing dataset, and the linear regression model was trained on the training dataset. The model was then tested on the testing dataset to evaluate its performance.

To test the effectiveness of implementing a convolutional neural network (CNN), the dataset was again split into a training dataset and testing dataset. Back Propagation (BP), General Regression Neural Network (GRNN), and CNN were implemented and their accuracies were compared.

To ensure the reproducibility of the results, the code used in the experimentation is available upon request.

### Calculating the Pearson’s Correlation Coefficient (PCC)

In order to investigate the impact of Covid-19 on the traffic system, we calculate Pearson’s correlation coefficient (ra) between the two variables Number of Covid-19 cases (NC) and Traffic Volume (TV) in six different countries with different traffic conditions as shown in Table 2.

**Table 2:** The r_a_ between the Number of Covid-19 cases (NC) and Traffic Volume (TV) in six different countries

Country	Population (2020)	ra
Egypt	102.3 million	-0.31028
UK	67.22 million	0.265033
US	329.5 million	0.026414
Italy	59.55 million	-0.33967
India	1.38 billion	-0.10206
France	67.39 million	-0.04302

From Table 1, it is shown that the results of calculating ra makes sense for each country depending on the number of population and the traffic.

### Calculating the Linear Regression (LR)

In order to predict the changes in the traffic volume from the number of the new cases of covid-19. We use Linear Regression (LR). The main goal of the linear regression (MLR) is to model the form of the relationship between the explanatory (independent) variables which are: (i) the traffic volume, and (ii) response (dependent) variable which corresponds to the number of new cases. The higher value of the R-square, the high relationship exists between dependent variables and independent variables. In our case of using the number of new cases and the traffic volume. In case of apply on the data set of Egypt the R-square = 0.096877107. The used equation from the data analysis to predict the predictive traffic volume (PTV) is PTV = 144.38—0.03NC, where NC is the number of covid cases.

**Fig. 4:**
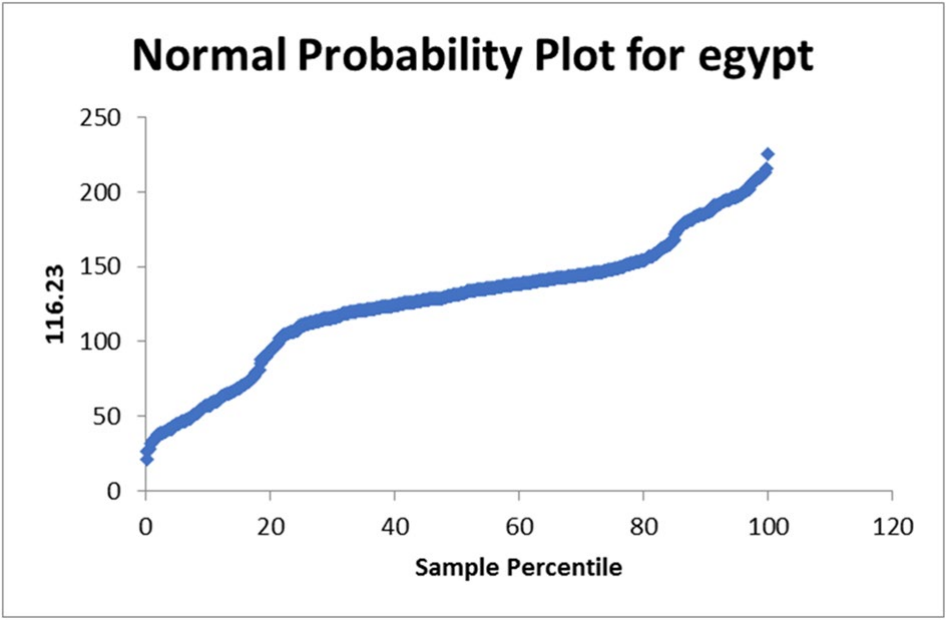
Normal Probability plot for Egypt

**Fig. 5:**
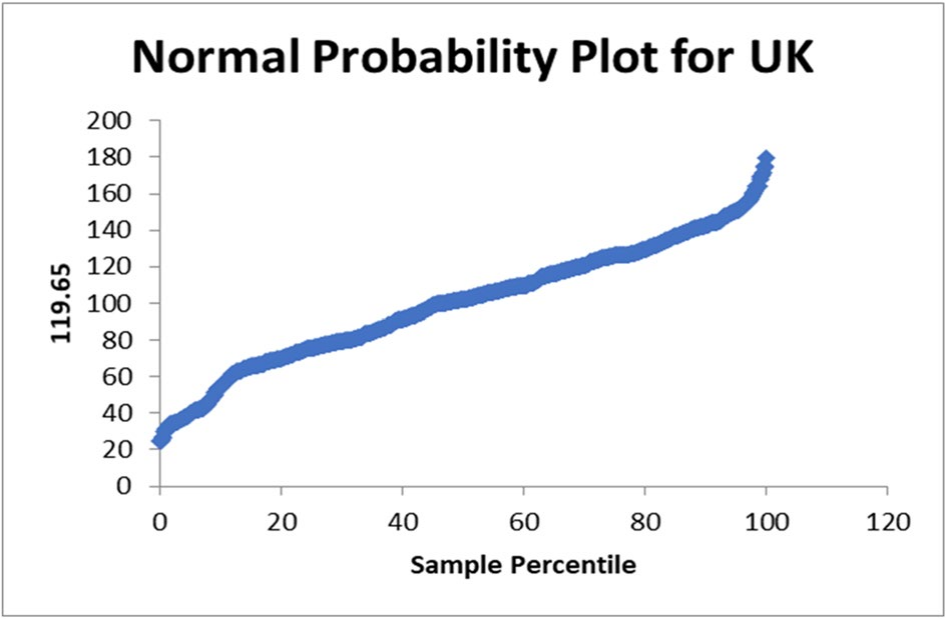
Normal Probability plot for United Kingdom

**Fig. 6:**
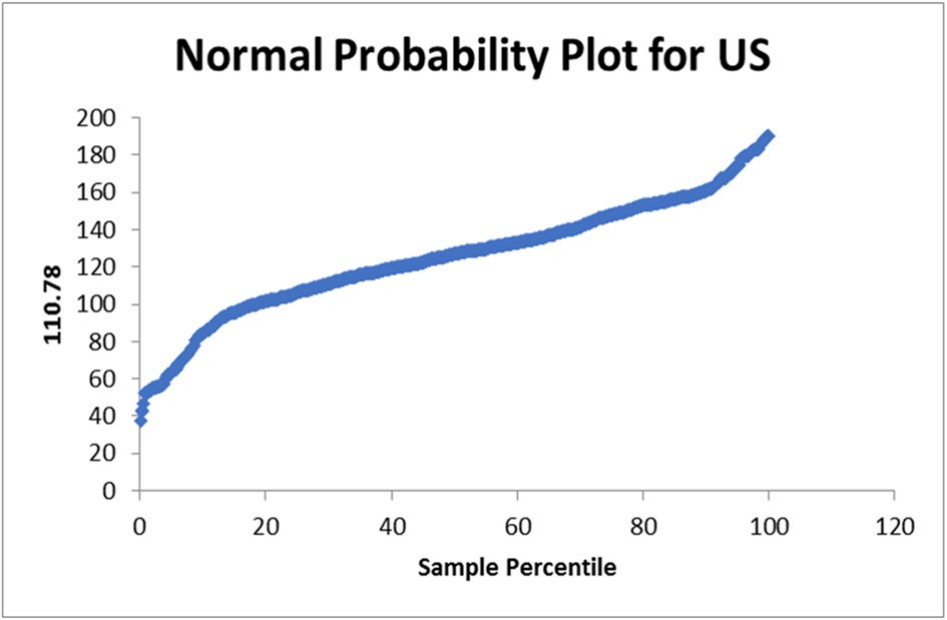
Normal Probability plot for United States

There normal probability plot for Egypt is shown in Fig. 4. In case of apply on the data set of United Kingdom the R-square = 0.070895526. The used equation from the data analysis is PTV = 91.5 + 0.005NC. The normal probability plot for United Kingdom is shown in Fig. 5. In case of apply on the data set of United States the R-square = 0.000653. The used equation from the data analysis is PTV = 124 + 0.0001NC. The normal probability plot for United States is shown in Fig. 6. In case of apply on the data set of Italy the R-square = 0.115389814.

**Fig. 7:**
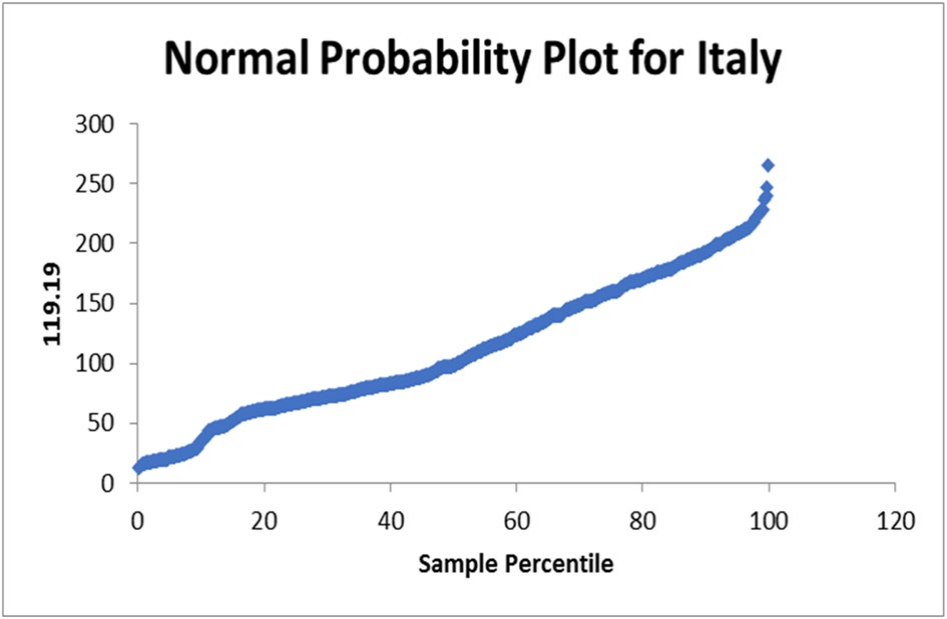
Normal Probability plot for Italy

**Fig. 8:**
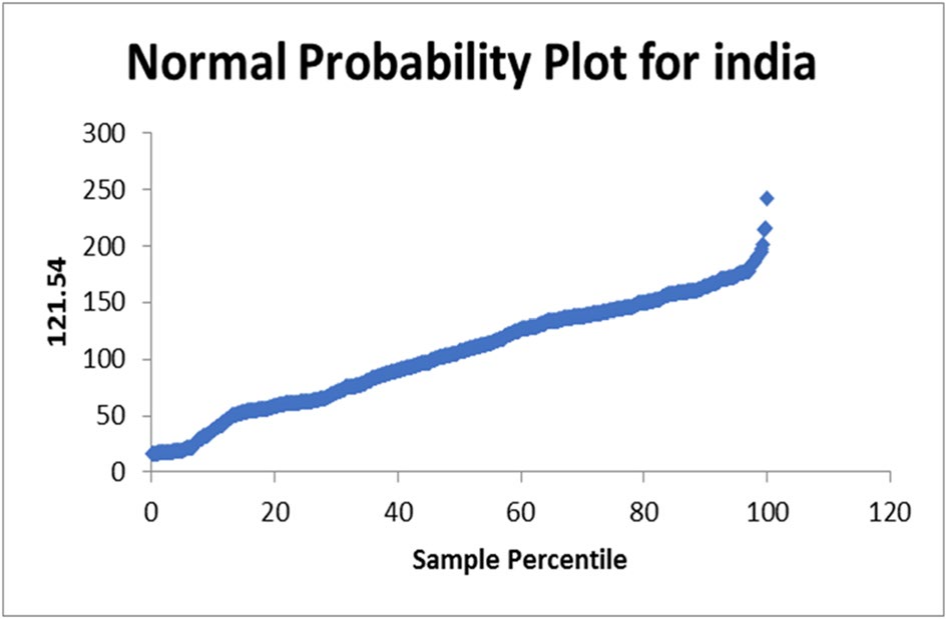
Normal Probability plot for India

**Fig. 9:**
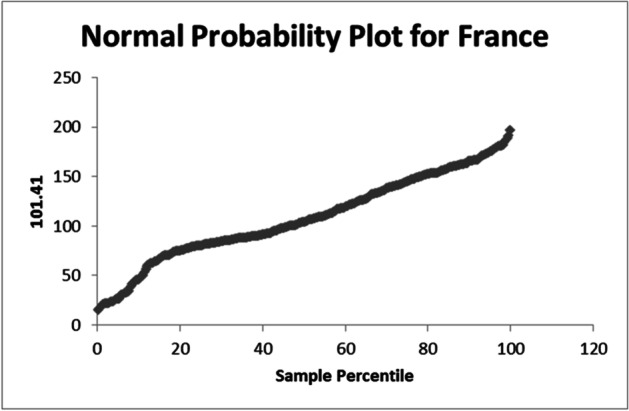
Normal Probability plot for France

The used equation from the data analysis is PTV = 128.4 – 0.002NC. The normal probability plot for Italy is shown in Fig. 7. In case of apply on the data set of India the R-square = 0.010334275. The used equation from the data analysis is PTV = 107.8—0.006NC. The normal probability plot for India is shown in Fig. 8. In case of apply on the data set of France the R-square = 0.001873129. The used equation from the data analysis is PTV = 109.8 – 0.0001NC. The normal probability plot for France is shown in Fig. 9.

### Results of Traffic Prediction Module (TPM)

After implementing TPM and calculating the accuracy, it is demonstrated that the accuracy is acceptable based on the magnitude of the realistic dataset utilized. The precision is 0.89. Before implementing Back Propagation (BP), General Regression Neural Network (GRNN), and CNN, we divide the used dataset into a training dataset and a testing dataset in order to evaluate the effect of CNN. Table 3 displays the values of Mean and Standard deviation.

**Table 3:** Accuracy of BP, GRNN, and CNN

Algorithm	Training Dataset		Testing Dataset	
	Mean	Standard deviation	Mean	Standard deviation
BP	17.73	5.35	7.35	3.19
GRNN	9.03	2.59	7.1	1.59
PNN	21.00	0.02	9.00	0.009

The results have proven that CNN has the best prediction accuracy.

### Results discussion

The results of implementing the proposed Traffic Prediction Technique (TPT) demonstrate its effectiveness in predicting the impact of the spread of COVID-19 virus on transportation. After implementing TPM and calculating the accuracy, it was found that the accuracy rate of 0.89 is acceptable given the size of the used dataset. This indicates that the proposed TPT is capable of accurately predicting traffic patterns during a pandemic.

To further test the effectiveness of the proposed TPT, the dataset was partitioned into a training dataset and testing dataset, and three different algorithms were implemented: Back Propagation (BP), General Regression Neural Network (GRNN), and Convolutional Neural Network (CNN). The results of the testing are presented in Table 3, which shows the mean and standard deviation values for each algorithm.

The results indicate that CNN has the highest accuracy rate compared to the other algorithms, with a mean of 21.00 for the training dataset and 9.00 for the testing dataset. This demonstrates the effectiveness of the proposed Traffic Prediction Module (TPM) that uses CNN for predicting the impact of COVID-19 on transportation.

Overall, the results of this study demonstrate the efficacy of the proposed TPT in predicting traffic patterns during a pandemic. By introducing a Traffic Prediction Module (TPM) that uses Convolutional Neural Network (CNN), the proposed TPT provides a novel and effective approach for predicting the impact of COVID-19 on transportation.

## Conclusions and future work

In conclusion, this research paper has successfully addressed the research gap by proposing a Traffic Prediction Technique (TPT) that incorporates a Traffic Prediction Module (TPM) using Convolutional Neural Network (CNN) to predict the impact of the COVID-19 pandemic on transportation. The methodology of impact calculation using Pearson’s Correlation Coefficient (PCC) and Linear Regression (LR) was also introduced. The simulation results from the five different networks demonstrated the effectiveness of the proposed TPT in predicting traffic impact accurately. Future work includes expanding the dataset to cover a longer period, incorporating more variables such as socioeconomic factors, and optimizing the CNN model to enhance its accuracy and efficiency. The use of CNN in the TPM module is a significant contribution to this research, as it has been proven to have a high accuracy rate in traffic prediction tasks. The potential of optimizing the CNN model for traffic prediction tasks is also worth exploring, as it could further improve the accuracy and efficiency of the proposed TPT. In the future, the proposed algorithm can be used with OCNN [27, 29–32, 34] and make use of Resnet [13].

## Data Availability

Data will be available on request.
